# Climate drivers of historic tree growth in the Vienna Woods and prediction of future performance of eight selected tree species

**DOI:** 10.1016/j.foreco.2026.123674

**Published:** 2026-06

**Authors:** Klaus Dolschak, Emilia Winter Artusio, Mathias Mayer, Michael Grabner, Michael Tatzber, Iftekhar U. Ahmed, Elisabeth Wächter, Isolde K. Berger, Pétra Berger, Wolfgang Wanek, Torsten W. Berger

**Affiliations:** a Institute of Forest Ecology, Department of Ecosystem Management, Climate and Biodiversity, BOKU University , Peter Jordan-Straße 82, Vienna 1190, Austria; b Institute of Wood Technology and Renewable Materials, Department of Natural Sciences and Sustainable Resources, BOKU University , Konrad Lorenz-Straße 24, Tulln an der Donau 3430, Austria; c Austrian Research Centre for Forests (BFW) , Department of Forest Protection, Unit of Air Pollution and Plant Analysis, Seckendorff-Gudent-Weg 8, Vienna 1131, Austria; d Centre for Microbiology and Environmental Systems Science, Division of Terrestrial Ecosystem Research, University of Vienna , Djerassiplatz 1, Vienna 1030, Austria; e Municipal Department 23 (Economy, Labor and Statistics) of the City of Vienna, Meiereistraße 7, Vienna 1020, Austria

**Keywords:** Forest water balance, Soil drought, Climate change, Dendrochronology, Growth factors, Drought stress indices

## Abstract

This study investigates the climate sensitivity and resilience of radial growth in eight coniferous and deciduous tree species in the Vienna Woods, Austria. Using dendrochronological methods, we analyzed tree-ring width data from 63 forest plots to assess growth responses to meteorological variability over the period 1933–2023. Historic climate records were used to develop a water balance model, from which we derived seasonal growth factors. Linear mixed effects models were applied to quantify species-specific relationships between tree-ring width and climatic conditions during the current and preceding two years. Tree-ring width responded not only to climatic conditions of the current growing season but also strongly to those of the previous year. Soil moisture and air temperature emerged as the principal drivers of radial growth, with soil moisture positively and temperature negatively affecting ring width. Climatic conditions during June-July of the current year exerted the strongest impact on ring formation. Using regional climate trends and projected air temperature and precipitation trajectories for Central Europe under RCP4.5 and RCP8.5, we forecast future growing conditions for the region. Both scenarios predict an extended growing season, increased transpiration demand, and heightened drought risk - more pronounced under RCP8.5. However, projected increases in precipitation partly offset the drought risk. By combining historical climate sensitivity of radial increment with future climate projections, we modelled expected tree-ring growth for eight tree species. Most species are predicted to experience notable declines in radial growth, with the strongest reductions in conifers, including European larch *(Larix decidua)*, Norway spruce *(Picea abies)*, Austrian pine *(Pinus nigra)* and Scots pine *(Pinus sylvestris)*. Deciduous species - Sycamore maple (*Acer pseudoplatanus)*, European beech (*Fagus sylvatica)*, and sessile oak (*Quercus petraea)* - show moderate declines. In contrast, Turkey oak *(Quercus cerris)* is projected to increase radial growth under future climate scenarios. These findings suggest that forest management in the Vienna Woods and adjacent regions should prioritize the promotion of warm- and drought-tolerant tree species such as *Quercus cerris* to enhance forest resilience and sustainability in the face of climate change.

## 1 Introduction

According to recent analyses, the mean annual temperature (MAT) in Austria has risen by 2.9 °C since the pre-industrial period. Austria is experiencing accelerated warming, leading to more frequent and severe heat extremes, reduced snow cover, and glacier retreat (Chimani et al., 2024). Higher temperatures are associated with an increased evaporative demand leading to an elevation of the drought stress risk (Senf et al., 2020). In parallel, rising temperatures have also led to an elongation of the growing season of the main deciduous tree species. A longer period of water consumption during the growing period alongside increased evapotranspiration rates can exacerbate drought stress, especially in broad-leaved trees (Dolschak et al., 2019; Meier et al., 2021). Coniferous species - on the other hand - might in theory respond positively to rising winter temperatures, due to their capability to maintain photosynthesis (Larcher, 2000). Besides varying responses on the interspecific level, dendrochronological records also point to different responses within different geographic populations of the same species.

For example, *Fagus sylvatica* - the dominant tree species in Central Europe - shows a pronounced north-south gradient in growth trends. While northern populations benefit from longer growing seasons due to warming (Pretzsch et al., 2023), southern populations face declining growth due to increased drought stress (Martinez del Castillo et al., 2022). *Quercus petraea* has been assumed to be less vulnerable to climate stress - such as higher temperatures and drought - than *Fagus sylvatica* (Leuschner et al., 2024). However, in Austria, tree-ring widths of *Quercus petraea* and *Quercus robur* (Pedunculate oak) were significantly reduced by water deficits, with the strongest growth-climate correlations observed for the current-season (Feb.-Apr.) climatic water balance (Garamszegi et al., 2025). Also, Radaković and Stajić (2021) found that the radial growth of *Quercus petraea* in northeastern Serbia was strongly reliant on summer precipitation, particularly in June and July, with latewood and total ring width showing the clearest response; temperature had no significant effect. In a study by Kostić et al. (2022), tree-ring width, stable carbon isotope ratio (δ^13^C), and intrinsic water use efficiency (iWUE) chronologies were developed for the period 1961–2000, focusing on two oak species - *Quercus robur* and *Quercus cerris* - in northwestern Serbia (Vojvodina province). The researchers examined the response of these species to meteorological data (temperature, precipitation, cloud cover), as well as drought events indicated by six meteorological drought indices and river water levels, to assess their drought tolerance and stress. The findings revealed that *Quercus cerris* exhibited better drought tolerance and lower drought stress compared to *Quercus robur*, evident through less negative δ^1 3^C values and lower iWUE.

Gadermaier et al. (2023) investigated the impact of soil water storage capacity and nutrient status on tree growth across 1562 forest sites in Styria, Austria. Their findings indicated that *Quercus* species - which are generally deep-rooting - significantly benefit from high water storage due to their access to deeper soil moisture, whereas shallow-rooting species like *Picea abies* rely more on upper soil layers. Considering the growth response of coniferous species. D’Andrea et al. (2023) found that the radial growth of *Picea abies* in Central Bohemia was primarily influenced by precipitation during the growing season, with little to no statistically significant correlation to temperature. This highlights the high sensitivity of *Picea abies* to drought - especially at lower elevations. Investigating *Pinus nigra* stands in a research area very close to the one in this work, Leal et al. (2008) reported a significant change in the sensitivity of tree growth rates to water availability in the late 20th century, with trees that were previously sensitive to spring-summer droughts showing reduced responsiveness. Tree-ring indices in the latter half of the 20th century exceeded expectations despite drought conditions, suggesting that water availability became less limiting to growth. This improvement was attributed to increased CO_2_ concentrations enhancing water-use efficiency in *Pinus nigra* in the Vienna basin, along with potential contributions from nitrogen deposition from the urban area improving nutrient availability. Overall, *Pinus nigra* is expected to benefit from climate change, with the potential to expand its habitat range towards the northeast (Klisz et al., 2023).

Climate projections for Lower Austria (ÖKS15; Chimani et al., 2016) indicate that - besides a projected rise in MAT - also mean annual precipitation (MAP) sums are likely to increase over the coming decades, with gains concentrated mainly in the dormant season (wetter winters). Summer precipitation is assumed to increase to a much smaller extent. Combined with rising potential evapotranspiration - driven by the projected temperature increase - it remains uncertain whether higher annual precipitation will compensate for elevated potential evapotranspiration, especially during critical early-summer periods. This uncertainty is particularly consequential in the Vienna Woods, which are located close to the eastern dry limit of *Fagus sylvatica* distribution (Bohn et al., 2007), where modest shifts in summer soil water availability could disproportionately affect the species’ performance and associated ecosystem services.

In this study, we focus on well-documented sites (Berger et al., 2016) and eight already well-established species (Winter Artusio et al., 2025) in the Vienna Woods, employing dendrochronological methods combined with water balance modelling to analyze their growth responses to changing climatic conditions. By modelling ring width as a function of meteorological growth factors, we aim to predict the future performance of these species under projected climate scenarios and state the three following hypotheses: (Hypothesis 1) Projected increases in MAP will not fully compensate for rising atmospheric evaporative demand, such that elevated transpiration demand will result in neutral to negative long-term effects on radial growth across most tree species. (Hypothesis 2) Seasonal conditions in the current growing season - especially June-July of the target year - explain more interannual variance in ring-width residuals than previous year conditions; moreover, within those key growing-season windows water availability is a stronger predictor of radial growth than air temperature. (Hypothesis 3) With the exception of *Pinus nigra*, coniferous species *(Larix decidua, Picea abies, Pinus sylvestris)* will exhibit stronger negative responses to elevated summer temperature and reduced summer soil moisture than deciduous species *(Acer pseudoplatanus, Fagus sylvatica, Quercus cerris, Quercus petraea*); among deciduous trees the *Quercus* species will display the most favorable response. This study aims to provide insights for local forest management. These findings might help improve management practices by identifying and promoting tree species that are more resilient to climate change, contributing to sustainable forest management in the Vienna Woods region.

## 2 Material and methods

### 2.1 Study area

**Fig. 1:**
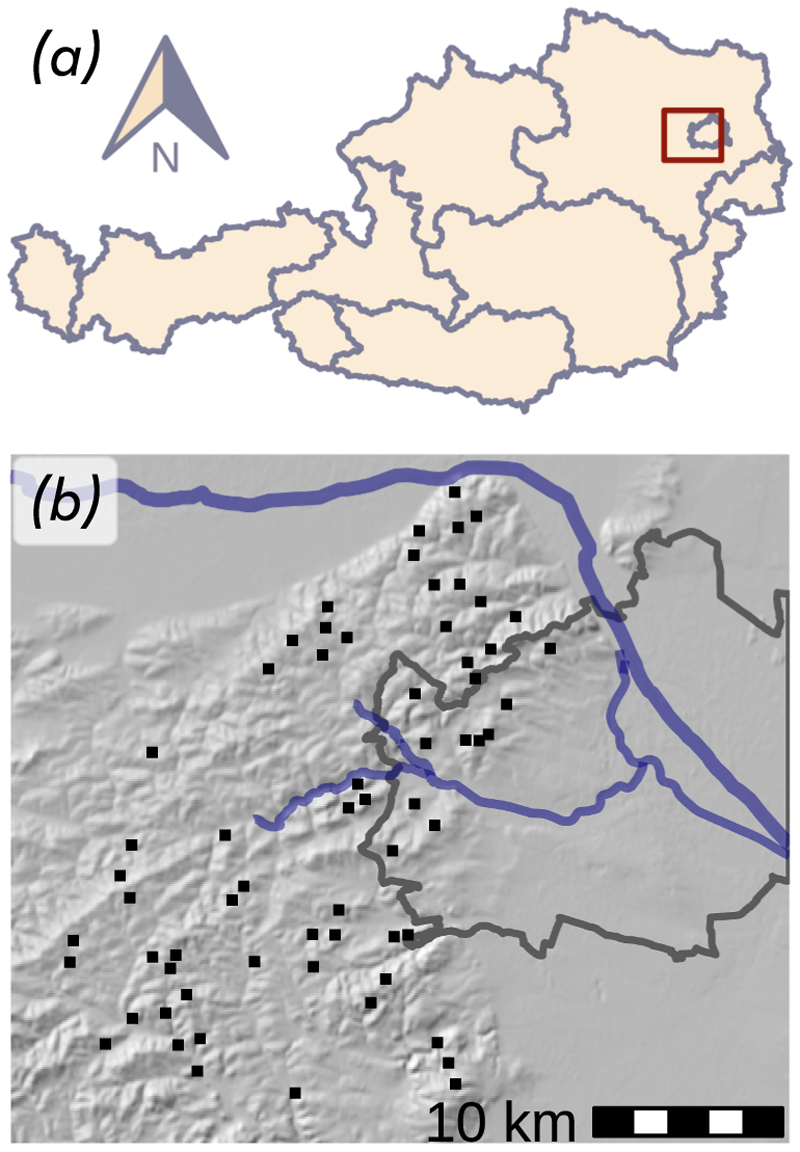
Research area in the Vienna Woods. (a) Location of the investigated sites in north-eastern eastern part of Austria. (b) All forest plots are located in the Vienna Woods. The gray outline indicates the municipal boundaries of Vienna. Out of 63 sites, eleven are situated within Vienna, while the remaining sites are located in Lower Austria. The blue line at the top represents the Danube River, while the blue line in the center marks the Wienfluss River along with one of its tributaries. The bedrock of the northerly part consists of Flysch sediment. Limestone forms the bedrock in the southerly part of the investigated area. All sites are beech stands, with different coniferous and deciduous species admixed.

The investigated forest plots are located in the Vienna Woods (Wienerwald), a lowland temperate forest region in close proximity to the City of Vienna, Austria (Fig. 1). The predominant bedrock is Flysch, a clay-rich marine sediment. Calcareous bedrock associated with the Northern Limestone Alps can be found in the southern part of the study area. The dominant soil types are Stagnosols and Cambisols (Schad, 2023). The elevation of the investigated plots ranges from 260 to 600 m above sea level. The mean annual precipitation varies between 600 and 900 mm, increasing from northeast to south-west. The mean annual temperature ranges from 8 to 10 °C depending on altitude. The dominant tree species in the investigated area is *Fagus sylvatica*. In autumn of 2022, 63 forest stands were sampled. In each stand *Fagus sylvatica* and co-occurring coniferous and deciduous species, i.e., *Acer pseudoplatanus, Larix decidua, Picea abies, Pinus nigra, Pinus sylvestris, Quercus cerris* and *Quercus petraea* (each with seven individuals per species and stand) were targeted for radial growth analysis. A detailed description of the investigated forest stand structure and metrics can be found in Winter Artusio et al. (2025) and Mayer et al. (2025).

### 2.2 Data sources

#### 2.2.1 Tree-ring chronologies

Sixty-three sites were sampled in autumn of 2022, with seven trees per site and species being sampled. Two cores per tree were taken at breast height. The trees’ ages ranged from 50 to 250 years. The cores were prepared for dendrochronological analysis by slicing the cross sections with a sharp knife and scanning them at 1200 dpi using a calibrated Epson Expression 10000XL. Ring widths were measured with the WinDendro 2022 system (Regent Instruments Inc., Quebec, Canada). Cross-dating was performed using Rinntech TSAP-Win (Rinn, 2003) to ensure accurate ring dating, and COFECHA (Holmes, 1983) was used to evaluate measurement quality. Cores that could not be reliably cross-dated due to defects (e.g., rot, breakage, reaction wood) were excluded. Measured pith-offsets were used to estimate the trees’ age. The focal cross-dated period finally covered 90 years and extended from 1933 to 2023. Further details of the used methods are given by Winter Artusio et al. (2025).

Tree age has a strong influence on tree-ring width (Gadermaier et al., 2024). Annual tree-ring growth declined non-linearly with age, levelling off only for stands older than 200 years; the age trend was comparable across species. In that work, they set up a GAMS model (General Algebraic Modeling System; Bussieck and Meeraus, 2004) and used tree age as a parameter. In our work, we developed a species-specific model to directly remove the age component. The average ring width by age (*w*_age_) is calculated as:

(1) $$w_{age\;}=\frac{p_{n}}{\;\text{age}\;+p_{s}}+w_{\min}-10k\times \text{ln}\left(1+\text{exp}\left(\left(p_{m}-\;\text{age}\;\right)\times 0.1\right)\right)$$

The helper variable *k* is calculated as:

(2) $$k=\frac{w_{\text{min}}-w_{50}}{p_{m}-50}$$

Parameters *p*_n_, *p*_s,_ *w*_50_, and *p*_m_ describe the shape of the transition of the age curve. *w*_min_ denotes the assumed minimum ring width.

The age-related component is then subtracted from the observed ring width (*w*_obs_). A remaining positive residual (*w*_res_) should correspond to favourable growing conditions and negative residuals to unfavourable environmental conditions.

(3) $$w_{\text{res}\;}=w_{\text{obs}\;}-w_{\text{age}\;}$$

The model was fitted using nonlinear least squares (nls) function in Base R (R Core Team, 2024).

#### 2.2.2 Meteorological data collection and processing

To analyze the influence of climatic factors on tree growth, meteorological data from multiple sources were compiled and processed, including both historical observations and future climate projections tailored to the study region.

For historical climate data, gridded daily temperature (minimum and maximum) and precipitation data from the Spartacus dataset (Hiebl and Frei, 2016) provided by GeoSphere Austria were used. The Spartacus dataset offers a 1 km × 1 km spatial resolution and covers the period from 1961 to present day. For this study, we set the temporal cut-off point to August 2025. Each forest stand was assigned to the corresponding grid cell in this dataset to obtain site-specific meteorological observations (minimum daily temperature, maximum daily temperature, daily precipitation sum). In addition, daily global radiation, wind speed, and relative humidity data were obtained from nearby meteorological stations (Wien-Mariabrunn, Wien-Hohe Warte) operated by GeoSphere Austria. These station-based measurements were used to extrapolate the data frame back to the year 1931 and to fill potential data gaps in the Spartacus dataset.

The Spartacus dataset does not offer information about global radiation. Instead, an estimate for the fraction of daytime sunshine is delivered. We used this value plus the calculated daylength (dependent on the day of the year plus geographical latitude) to derive an estimate from observed global radiation at the meteorological station Wien-Hohe Warte. A similar approach was applied to derive wind speed and relative humidity.

**Fig. 2:**
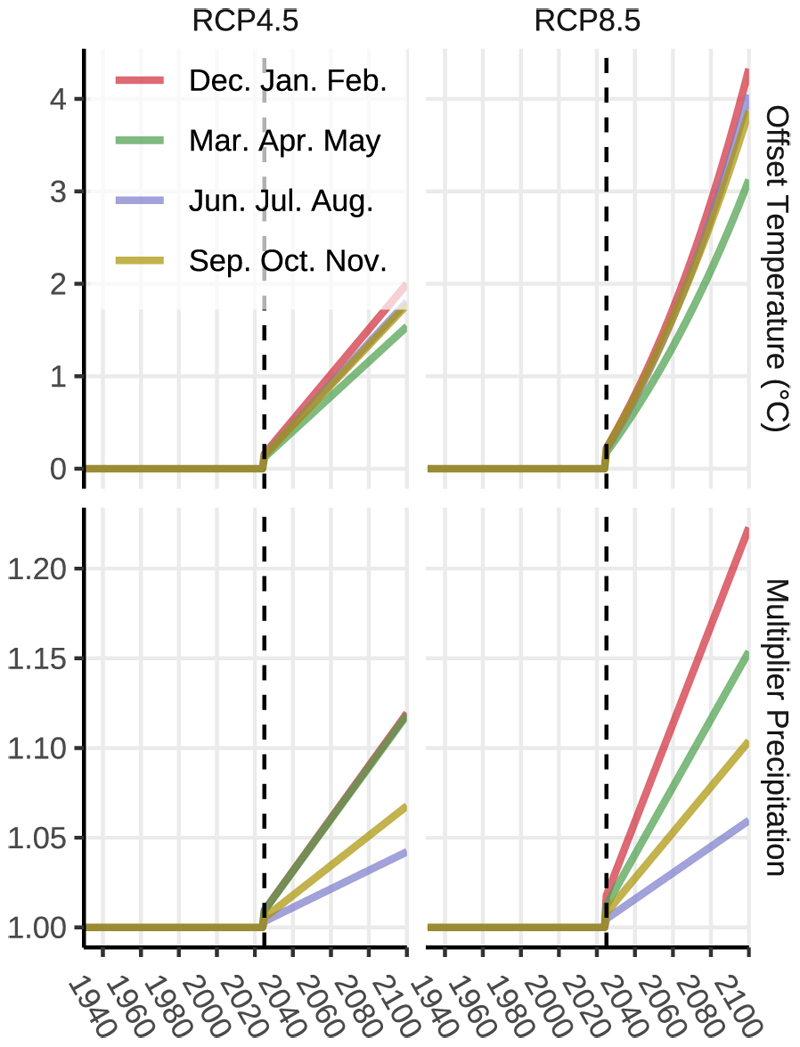
Seasonal modifiers of air temperature and precipitation derived from the ÖKS15 scenarios. For the baseline climate extension, we utilize historic weather data from 2001 to 2020 to create a 20-year loop of daily weather records extending until the year 2100. For temperature, a seasonal offset is applied; daily precipitation is adjusted by being multiplied by a seasonal precipitation factor. In the ÖKS15 scenarios, it is posited that winter temperatures and winter precipitation are more significantly impacted by climate change than summer conditions. In the more extreme RCP8.5 scenario, it is projected that temperatures will increase by approximately 4° C by the end of the century, with a less pronounced rise in summer months (about 3° C). Additionally, for the RCP8.5 scenario, winter months are expected to see an increase in precipitation of around 20%, while only a 5% increase is anticipated for summer months.

To model future climatic conditions and their effect on tree growth, climate projections were applied based on the ÖKS15 dataset (Chimani et al., 2016), which provides downscaled regional climate scenarios for northeastern Austria. Three climate scenarios were considered. A baseline (constant climate) scenario was created by looping historical meteorological data from the most recent 7305 days (20 years from January 2001 to December 2020) and repeating it from September 2025 to December 2100, maintaining stable climatic conditions over time. We intentionally excluded 2024 from the looped dataset because an extreme precipitation outlier in September 2024 could skew results. In addition to the baseline scenario, two climate change scenarios were used: RCP4.5 (representing a scenario with moderate greenhouse gas reductions) and RCP8.5 (a business-as-usual emission scenario). Both RCP scenarios project rising temperatures, with the warming trend being more pronounced in RCP8.5. Interestingly, besides rising temperatures, both scenarios’ projections also postulate an increase in precipitation. Seasonal adjustments were applied to daily precipitation and temperature. A seasonal multiplier was calculated to modify baseline precipitation values based on projected trends in the ÖKS15 dataset, while a seasonal offset was applied to the daily minimum and maximum temperatures to reflect expected warming trends (Fig. 2). This way, a continuous timeline of daily meteorological data was generated for each forest stand, covering the full modelling period from 1931 to 2100.

### 2.3 Modelling

#### 2.3.1 Fagus sylvatica phenology

To account for climate-change-induced shifts in the length of the growing season, we implemented the model presented by Dolschak et al. (2019), in which the onset of the growing season in *Fagus sylvatica* (leaf unfolding; LU; BBCH-11 according to Meier (2018) is modelled as the cumulative product of daily air temperature (above a threshold) and a daylength-dependent photoperiodic term (Blümel and Chmielewski, 2012). Leaf unfolding is triggered when the cumulative forcing exceeds a defined threshold.

The end of the growing season (leaf coloring; LC; BBCH-94 according to Meier (2018) is modelled using a temperature-based approach that considers two time-intervals: (i) late spring and (ii) late summer to early autumn. According to Estrella and Menzel (2006) lower temperatures in late spring and higher temperatures in late summer are associated with a delay in the end of the growing season. In our model, the temperature in the first interval has a negative effect on growing season length, whereas high temperatures during the second interval delay LC. The model was fitted to observations of BBCH-11 and BBCH-94 for *Fagus sylvatica* from the PEP725 database (Templ et al., 2018).

#### 2.3.2 Water balance model

To assess tree growth factors derived from meteorological conditions, we set up a water balance model (WBM) for each forest stand. The WBM, which runs on a daily time step, uses standard meteorological data on a daily basis as input. Time series of minimum, mean and maximum temperature, the daily averages of relative humidity, global radiation, and wind speed at 2 m above ground, as well as the observed 24 h precipitation sum are required. In addition, information about phenological stages, e. g., the start and the end of the growing season is required. An exhaustive formulation of the WBM can be found in the appendix of Dolschak et al. (2019). The model was parameterized on a timeline of observed soil moisture at the Level II plot Klausen-Leopoldsdorf, located in the Vienna Woods (48.120 N, 16.047 E), at 510 m a.s.l. For a detailed site description see Neumann et al. (2001). The research site is a pure *Fagus sylvatica* stand, and shares soil characteristics with the forest stands, investigated in this work. Disregarding inter-site variability, the WBM was applied to all sites using the same parameterization as for the Klausen-Leopoldsdorf site. We consider the beech-derived parameterization justified because all stands are dominated by *Fagus sylvatica*. The fact that the study sites exhibit very uniform soil types (stagnic cambisols to stagnosols) further minimizes inter-site soil-hydraulic variability.

Accounting for local topographic influences, incoming daily global radiation was adjusted for each individual plot by incorporating information on slope and aspect, using the approach proposed by Tian et al. (2001). The model was run for historic conditions until the present day. For future conditions three climate change scenarios were applied, presented in Section 2.2.2.

#### 2.3.3 Definition of growth factors

To quantify the influence of meteorological conditions on radial growth, a set of key environmental variables - referred to as growth factors - was defined: (i) *mean temperature*, representing the average air temperature over the respective period; (ii) *actual transpiration sum*, defined as the total amount of water lost through transpiration during the observed period, reflecting vegetation water use; (iii) *climatic water balance*, calculated as the difference between observed precipitation and actual modelled transpiration, providing a measure of net water availability; (iv) *transpiration deficit sum*, describing the difference between actual (modelled) and potential transpiration, with the latter estimated using the FAO Penman-Monteith equation (Allen et al., 1998), serving as an indicator of plant water stress; (v) *average modelled soil moisture*, representing the mean soil moisture content over the respective period; (vi) *number of days with REW (relative extractable water)* < *0.4*, capturing periods of severe soil water stress; and (vii) *number of days with REW* < *0.7*, indicating episodes of - at least - mild water stress. REW is defined as the relative soil moisture content (θ), scaled between 0 at the wilting point (*θ*_wp_) and 1 at the soil’s water holding capacity (*θ*_hc_).

(4) $$\text{REW}=\frac{\theta -\theta_{\text{wp}}}{\theta_{\text{hc}}-\theta_{\text{wp}}}$$

Following Granier et al. (1999), a threshold of 0.4 marks the onset of severe water deficit. In this work, we introduce a second threshold level of 0.7 corresponding to (at least) mild water deficit conditions. For both thresholds, we calculate the number of days on which the respective threshold was undershot in the designated period. All growth factors were standardized on a seasonal basis (mean = 0, standard deviation = 1) to enable direct comparison of the relative influence of each variable within the regression model.

Several cut-points in the definition of seasons were tested. The best results were achieved by splitting the growing season (here defined as April to September) into three blocks of two-month length. The remaining dormant season was split into two blocks of three-month length. Seasons were defined as the following periods: January to March, April to May, June to July, August to September, and October to December. In addition to the calculated growth factors of the current year (denoted as ‘act’), the influence of conditions from the previous year (‘-1’) and the year before that (‘-2’) were also considered. These temporal divisions allowed us to assess how both current and lagged climatic factors - such as calculated transpiration deficit and modelled soil moisture - affect the variation in the tree-ring width residual of the current year. We assume tree-ring growth is completed by the end of September; therefore, for the target year the period of consideration also ends in September.

#### 2.3.4 Mixed effects modelling

To investigate the relationship between tree growth and climatic conditions, time series (1933–2022) of ring width residuals were calculated individually for each tree species and site (N = 7, per species and site), including eight tree species; *Fagus sylvatica* occurred at all 63 sites. The Vienna Woods are dominated by *Fagus sylvatica* stands. Hence, for some species it was hard to find enough sample trees. However, the number of sample trees in this work roughly reflects the fraction it makes up in the species distribution of the Vienna Woods. *Acer pseudoplatanus* was only present at one site, so only 7 trees could be probed. This is below a minimum of 12–15 sample trees per site advised by Cherubini et al. (2020). As it is hard to disentangle site- and climate effects, the results for this species must be interpreted with caution. All other species were present at - at least - 4 sites, yielding a minimum of 28 trees per species, making the results much more reliable.

Growth factors were calculated by season, site and year and subsequently standardized on a seasonal basis, to allow comparison of the effect strengths of the coefficients in the regression model. To avoid multicollinearity, a preselection step was applied, allowing only one growth factor per season from each of two categories: (1) water (transpiration sum, transpiration deficit, climatic water balance, mean soil moisture, days below REW ≤ 0.4 and REW ≤ 0.7), and (2) temperature (mean temperature).

Linear mixed-effects regression models (LME) were then fitted individually for each tree species, with site included as a random effect, to account for spatial variation. Model selection followed a stepwise variable removal approach, continuing until no further reduction in the Akaike Information Criterion (AIC; Akaike, 1973) was achieved. Statistical analyses and visualizations were conducted in R (R Core Team, 2024) using the ‘nlme’ package (Pinheiro and Bates, 2000). The final model was applied to climate change scenario data to assess effects of potential future growing conditions on radial growth of the studied tree species.

## 3 Results

### 3.1 Predictions of phenological changes

**Fig. 3:**
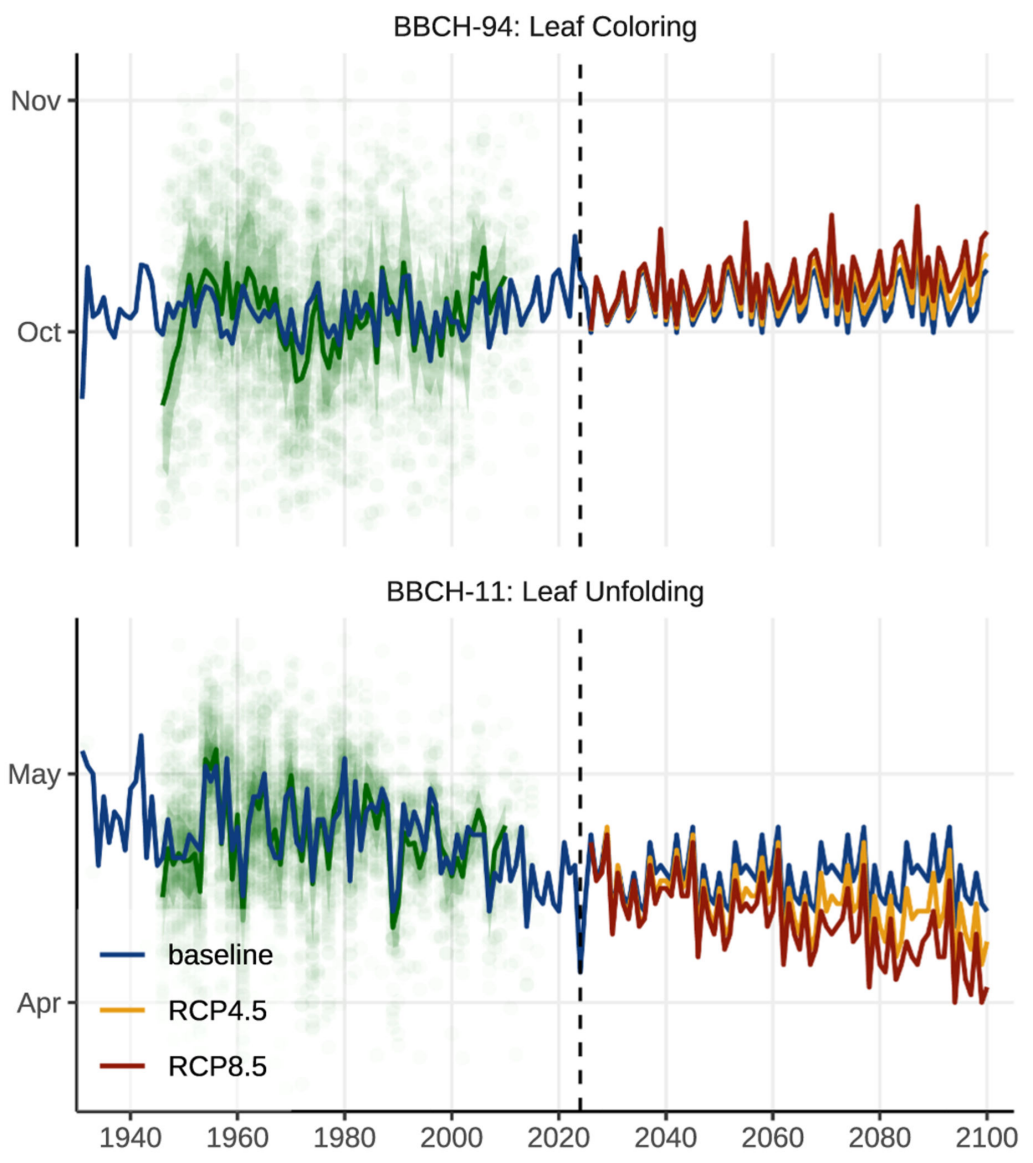
Observed and modelled phenological changes. The model was fitted to an annual mean (green line) of observed single events (green dots) retrieved from the PEP725 database (Templ et al., 2018). Observed events display high interannual plasticity. Nevertheless, a clear trend to earlier onset and later end of the growing season is visible in the data (compare: Dolschak et al., 2019). Both climate change expansions point to an elongation of the growing season until the end of the century by about two weeks (compared to the baseline scenario), whereas the change is more pronounced at the beginning of the growing season. An elongation of the growing season might exacerbate the drought stress risk due to higher water consumption, mainly at the first half of the growing season.

Phenological modelling indicates a lengthening of the growing season under both RCP4.5 and RCP8.5 (Fig. 3). By 2081–2100, the growing season is projected to be approximately seven days longer under RCP4.5 and about fourteen days longer under RCP8.5 compared with the baseline. This change is driven by an earlier onset of leaf unfolding by roughly five days (RCP4.5) to ten days (RCP8.5) and a later senescence by about two days (RCP4.5) to four days (RCP8.5).

### 3.2 Growth factors

**Fig. 4:**
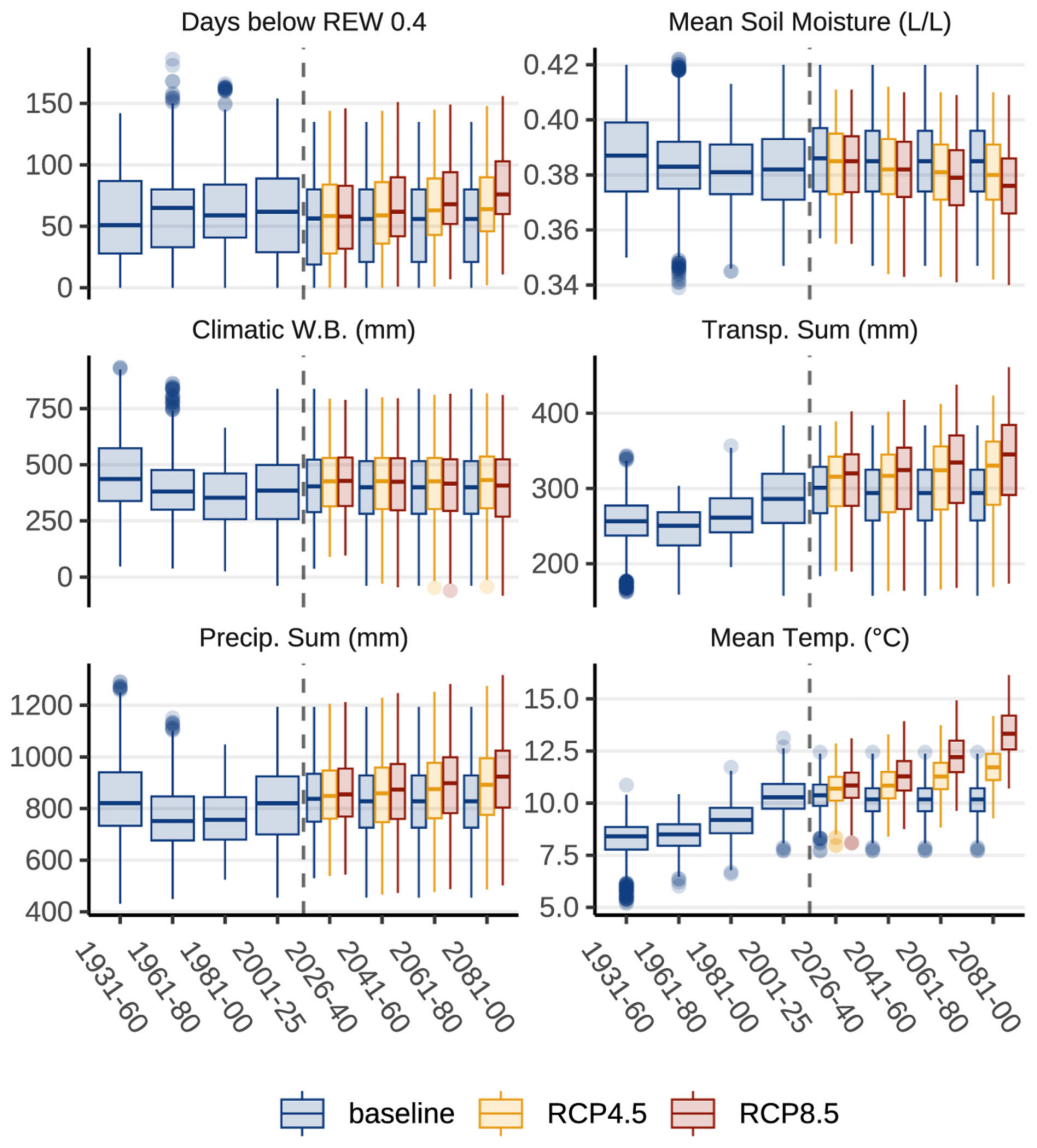
Modelled annual growth factors for the investigated climate change scenarios. Growth factors, aggregated over the full year and displayed for 20-year periods. The baseline weather timeline from 2026 on consists of looped data from 2001 to 2020. Therefore, no changes are visible in the future timeframe from 2026 to 2100. In the RCP8.5 scenario, increased transpiration causes average soil moisture to decrease, leading to more severe water stress (indicated by a rise in days with REW ≤ 0.4) that increases from approximately 50 to 70 days per year compared to the baseline scenario. These effects are less pronounced in the RCP4.5 scenario. Drought stress is partially mitigated by increased annual precipitating sums in the climate change scenarios.

**Fig. 5:**
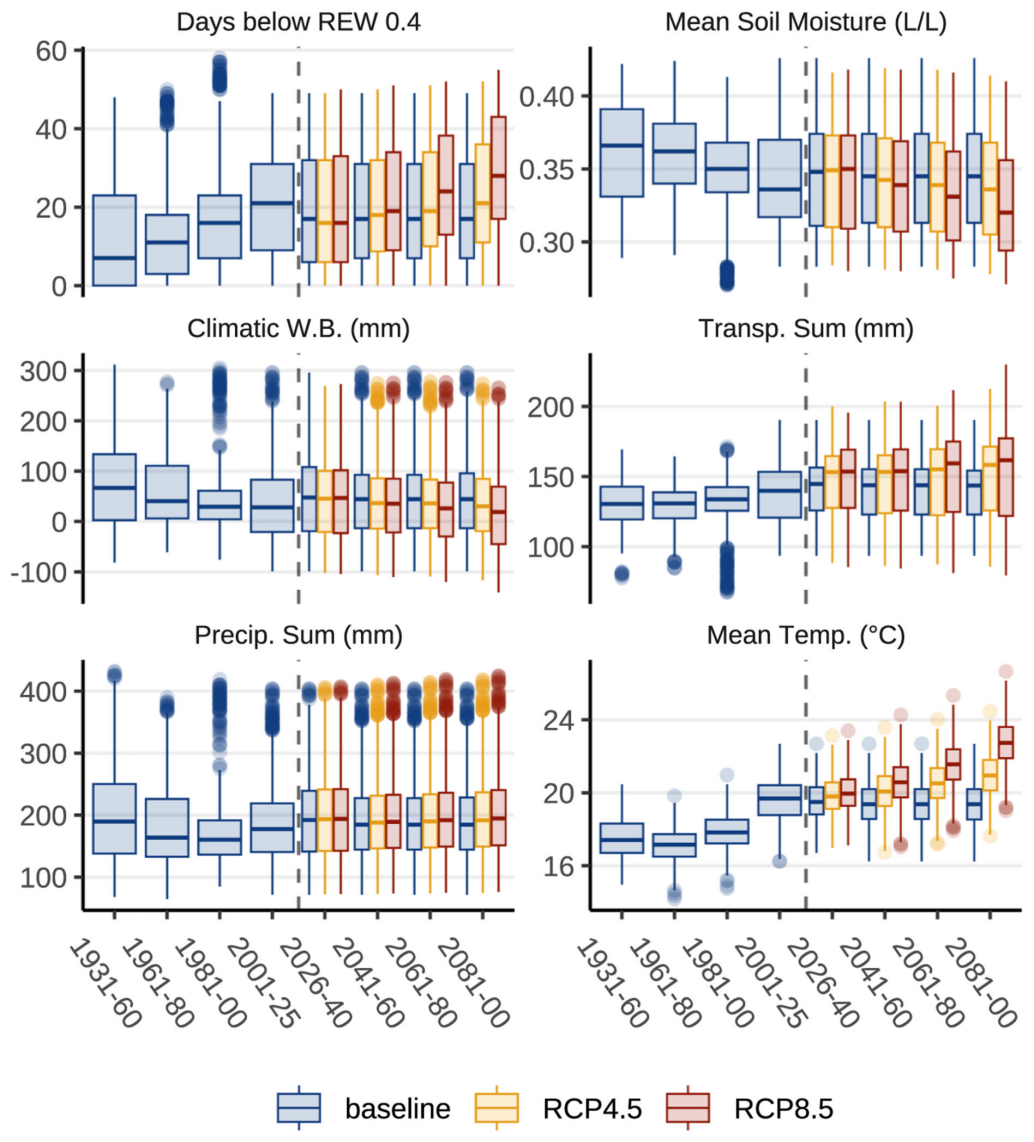
Modelled growth factors for season June - July for the investigated climate change scenarios. The period from June to July is deemed critical for tree ring formation. Elevated temperatures in the climate change scenarios contribute to increased transpiration sums, depleting soil moisture, therefore exacerbating water stress, as indicated by an increase in days of severe water stress (REW ≤ 0.4) from an average of 18 (baseline) to 30 days (RCP8.5) - nearly half of the days within this two-month period. In contrast to rising winter precipitation sums, summer precipitation levels exhibit only a minor increase in the climate change scenarios, resulting in, only minimal mitigation effects from higher rainfall.

Water balance modelling points to rising annual transpiration sums by ~21%, from approximately 290 to 350 mm until 2100 in the RCP8.5 scenario (Fig. 4). In our simulation, increased transpiration also leads to slightly drier soils. The transpiration effect is only partially mitigated by increased precipitation sums in the climate change scenarios. The annual climatic water balance displays only minor changes in the climate change scenarios until the end of the century. Rising annual precipitation sums in the RCP4.5 and RCP8.5 projections are primarily driven by rising winter precipitation. The annual number of days with severe water stress (REW ≤ 0.4) is assumed to increase from 50 to 65 until the end of the century in the RCP8.5 scenario. Because our work suggests that growth conditions during June and July have the highest impact on ring formation, similar graphs are plotted in Fig. 5 for this specific season.

### 3.3 Age-width relationship

**Fig. 6:**
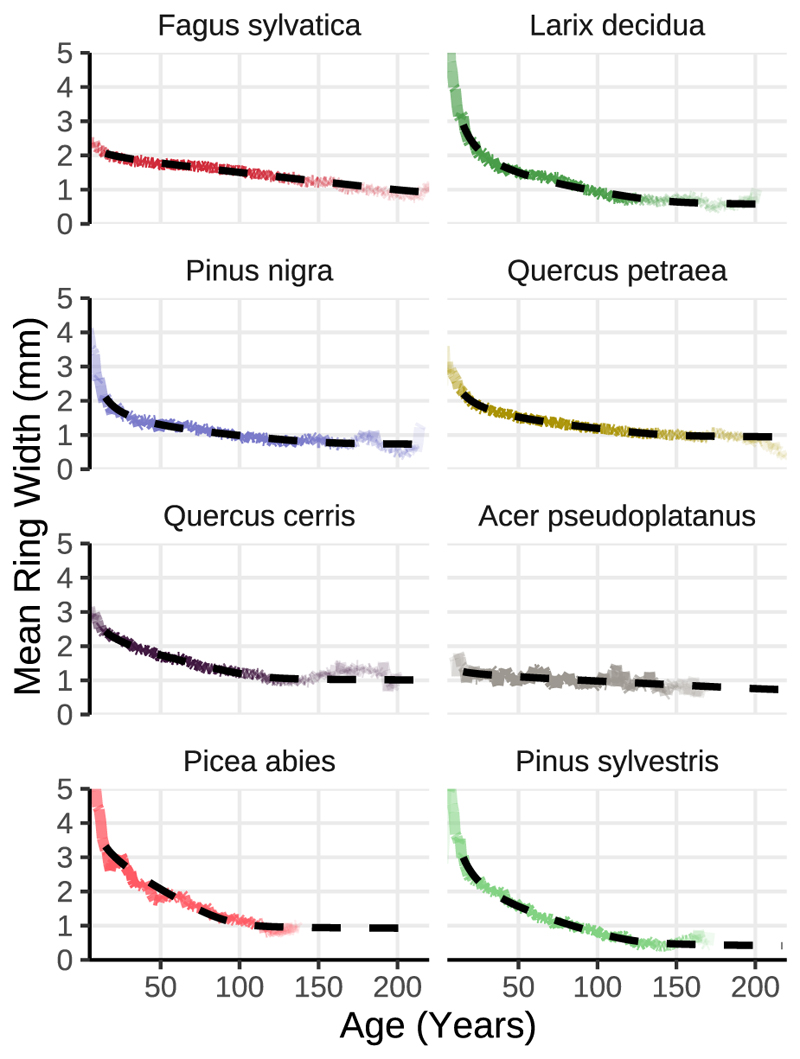
Observed and modelled relationship between tree age and ring width. Growth patterns vary during tree maturation. The coloured lines denote the observed average ring width for a given age, while the dashed line represents the species-specific model fit. We postulate that the residuals between the modelled and observed values depict the portion of ring width influenced by site-specific factors and local environmental conditions, such as weather conditions and drought periods.

The age-width model was fitted to the average age dependency of tree-ring width per tree species utilizing the ‘nls’ procedure (Fig. 6). The corresponding optimised parameter values are displayed in Table 1. Overall, the model fit (adjusted r^2^) ranged between 0.90 and 0.98, except being 0.28 for *Acer pseudoplatanus*. This somehow poor fit can be attributed to the small sample size (n = 7). The tree species was only present on one forest site. Therefore, all results for *Acer pseudoplatanus* must be interpreted with caution.

### 3.4 Tree-ring residual modelling

**Fig. 7:**
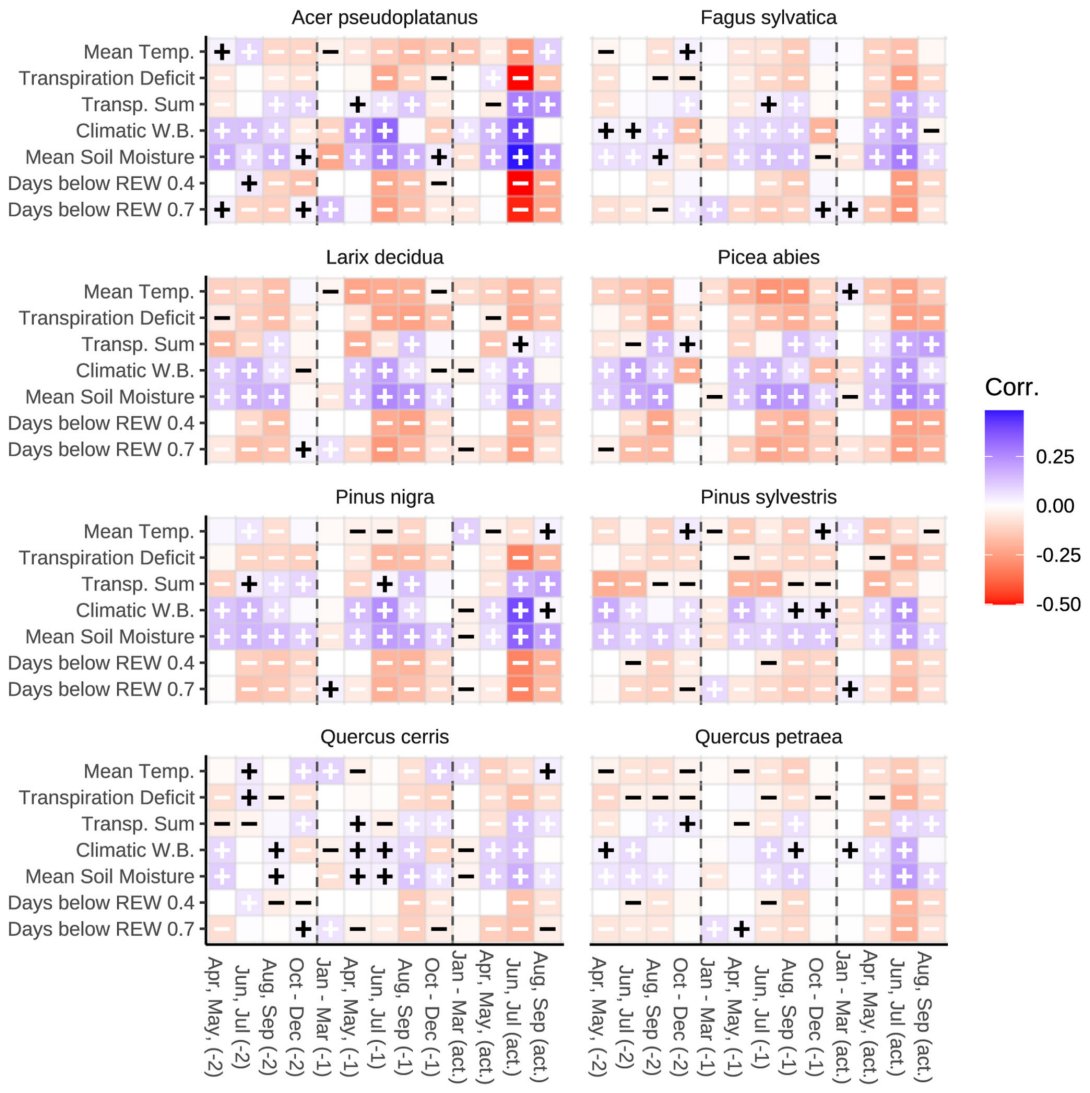
Correlation between seasonal growth factor and ring width residual. Growth factors were aggregated site-wisely and seasonally and calculated for (‘act’) the target year (year of tree ring formation), (‘-1’) the previous year, and (‘-2’) the year before that, as past conditions might also influence current growth. Seasonal periods were defined as follows: January to March, April to May, June to July, August to September, and October to December. Given that tree ring development is presumed to be complete by the end of September, the analysis timeframe for the target year also concludes with September. Subsequently, Pearson correlation coefficients were computed between the ring residuals and the growth factors. Blue fields indicate a positive correlation between the growth factor and the residual, while red fields indicate a negative correlation. The more intense the colour, the stronger the correlation. Across all tree species, it becomes evident that the growth conditions in the current year, especially during the growing season of June to July, exert the strongest influence on ring width. As expected, the correlations are significantly more pronounced during the summer months than outside the growing season. Soil moisture consistently correlates positively with growth, underscoring the importance of water availability for forest productivity. In contrast, temperature exhibits an opposite effect: warmer conditions are associated with smaller ring widths in the target year. *Quercus cerris* is the only exception here: Increased temperatures, especially during the period outside the growing season, lead to wider growth rings. This indicates a possible suitability concerning a warming climate.

Fig. 7 illustrates the relationship between seasonal growth factors and tree-ring residuals, displayed along the x-axis (seasons) and y-axis (growth factors). Each point on the graph represents a combination of growth factors and corresponding seasons, which are then input into the mixed effects model. The growth conditions in the current year, particularly in the season from June to July, had the most significant impact on ring width, indicated in darkest coloring of the tiles in Fig. 7, across all tree species and possible predictors. As anticipated, the correlations were much stronger in the summer months compared to other times of the year. Among all species, soil moisture showed a consistent positive correlation with growth, highlighting the critical role of water availability for forest productivity. Conversely, temperature had a negative effect, and higher temperatures were linked to reduced ring widths in the target year. This trend was contrasted by *Quercus cerris*, where radial growth exhibited a positive correlation with dormant season temperature from October to March.

**Fig. 8:**
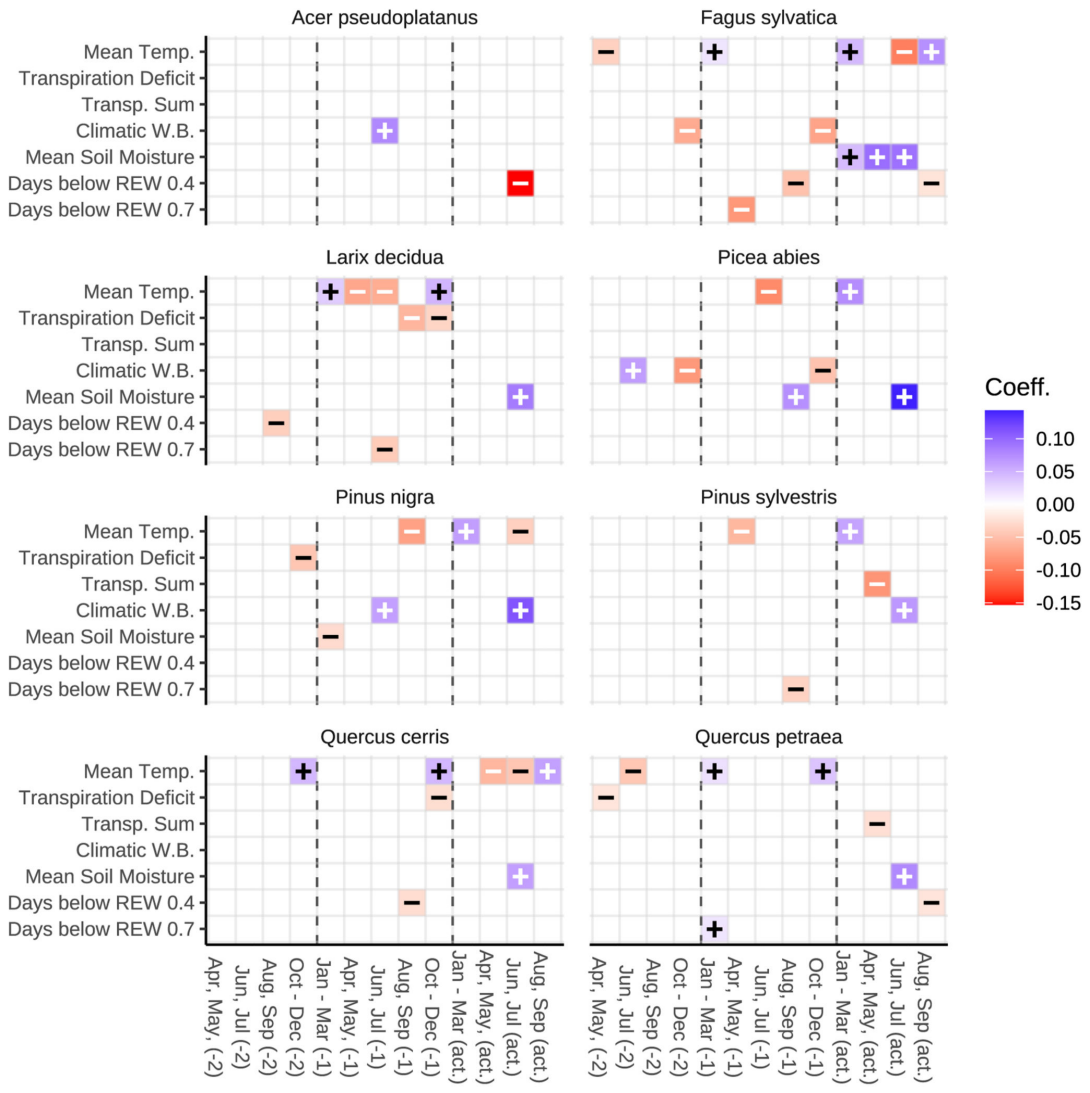
Remaining predictors in the mixed effects model. Growth factors were standardized for direct comparison of coefficient strength. The model was simplified by sequentially removing climatic predictors until further AIC reduction was no longer possible. Models with fewer data points had fewer predictors. For example, *Acer pseudoplatanus* was present only at a single site with a timeline of 90 years (90 data points), resulting in a model with only two predictors. In contrast, *Fagus sylvatica* was observed across all 63 sites, allowing for a more complex model structure with additional predictors. Predictors for all species can be viewed in Table 3. Stepwise variable reduction for *Acer pseudoplatanus* (single site, 90 yearly observations) lead two predictors remaining: (1) June-July climatic water balance of the year prior to ring formation, and (2) June-July drought days of the current year. *Fagus sylvatica* was observed across all 63 sites allowing for a more complex model structure with 13 growth factors as predictors. Most important were conditions in June – July of the year of ring formation. Temperature had a negative and soil moisture a positive effect on ring formation. Soil moisture (from Jan. to Jul.) in the year of ring formation had a positive effect on growth.

**Fig. 9:**
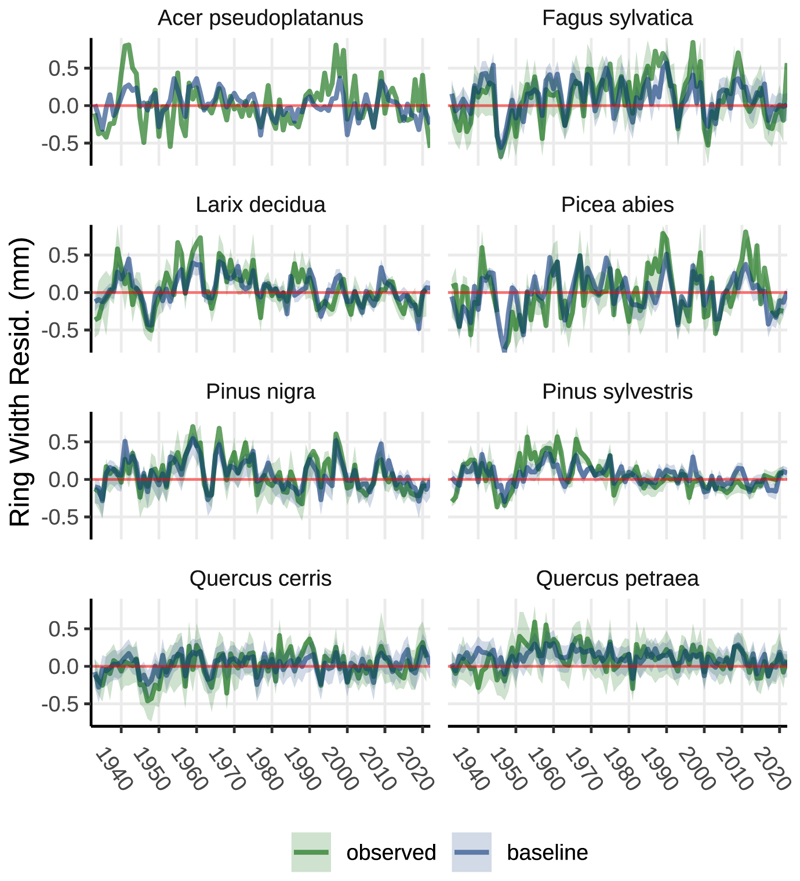
Observed and modelled (baseline climate) annual ring width residual for 8 tree species. The coloured lines represent observed (green) and modelled (blue) annual mean values. The green and blue ribbons represent the 25 and 75% percentiles of oberserved and modelled ring width residuals. The LME model offers a good fit for the temporal dynamic of the annual mean with r^2^ values ranging from 0.35 *(Acer pseudoplatanus)* to 0.70 *(Pinus nigra)*.

In Fig. 8, the remaining predictors in the mixed effects model are displayed. Models with fewer data points contained fewer predictors. For instance, *Acer pseudoplatanus* was only present at a single site with a 90-year timeline (90 data points), resulting in a model with just two predictors. In contrast, *Fagus sylvatica* was observed across all 63 sites, allowing for a more complex model structure with additional predictors. Species specific model performance is given in Table 2. Optimized predictor values for all species are listed in Table 3. Fig. 9 displays a comparison of observed and modelled annual mean ring width chronologies for all tree species. Our modelling approach offered a good fit for the observed temporal dynamics of tree-ring width, with r^2^ values ranging from 0.35 *(Acer pseudoplatanus)* to 0.70 *(Pinus nigra)*.

Below we describe the climate sensitivity and projected behavior of radial growth of the tree species in alphabetical order.

**Fig. 10:**
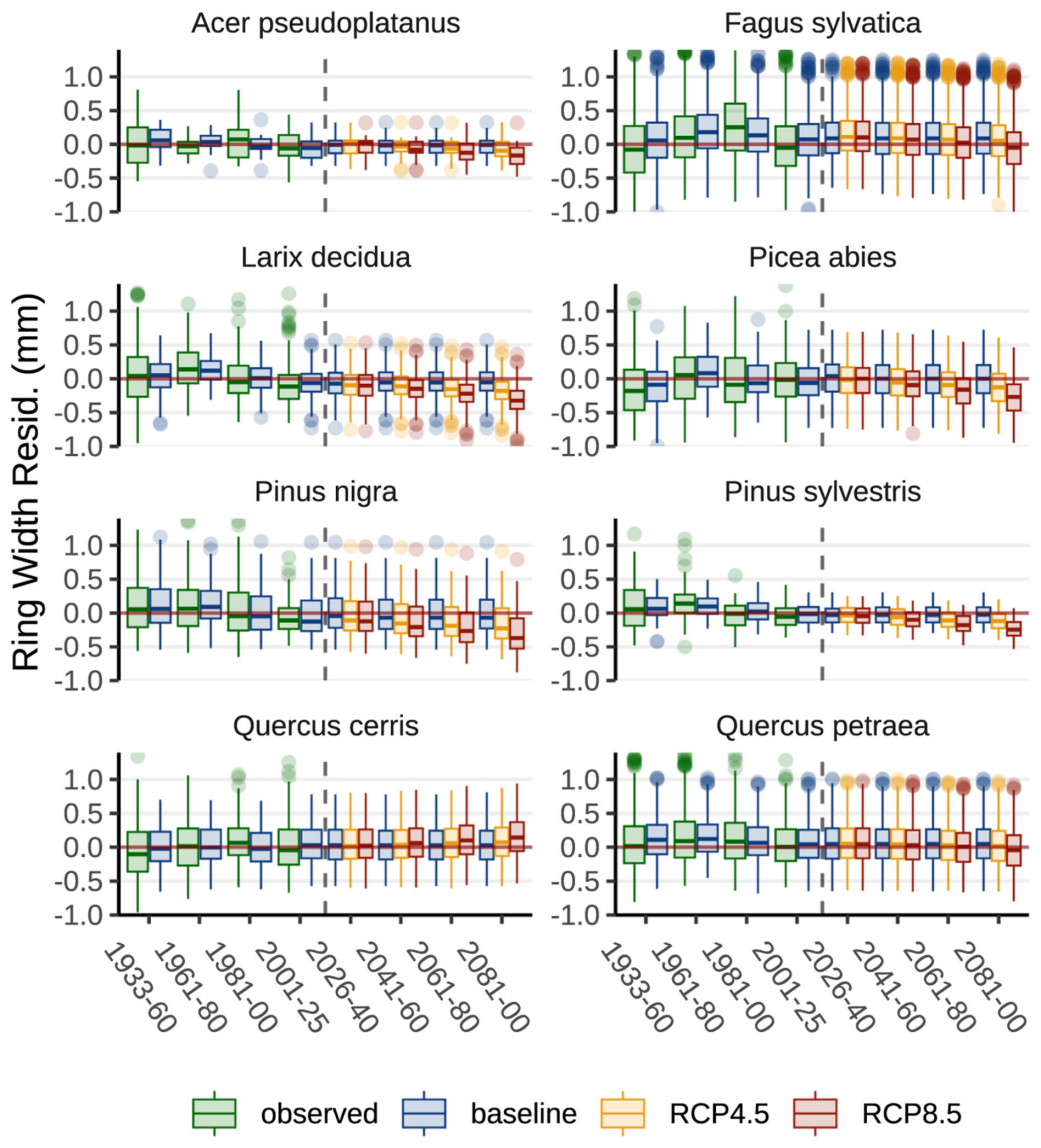
Annual ring width residuals for eight tree species. All species, except one, show a decline in average ring width residuals in the climate change scenarios by the century’s end. For the period 2081–2100, growth declines in the RCP8.5 scenario (compared to baseline) are as follows: *Pinus nigra*: −0.29 mm, *Larix decidua*: −0.27 mm, *Picea abies*: −0.26 mm, *Pinus sylvestris*: −0.21 mm, *Fagus sylvatica*: −0.15 mm, *Acer pseudoplatanus*: −0.14 mm and *Quercus petraea*: −0.09 mm. Notably, *Quercus cerris* demonstrates a slight increase of approximately +0.12 mm in average ring width residuals by century’s end in the RCP8.5 scenario, mainly due to positive responses to higher temperatures during late summer of the actual year, and late autumn of the preceding years.

For *Acer pseudoplatanus* the final model retained only two predictors: (i) The climatic water balance during June and July of the year preceding ring formation, and (ii) the number of drought days in June and July of the current growth year (Fig. 8). All other climatic predictors were excluded during model selection. Our simulation points towards a moderate decline of the mean annual ring width residual for the far future (2081–2100) of –0.14 mm (RCP8.5 scenario vs baseline; Fig. 10).

*Fagus sylvatica* was present across all 63 sites allowing for a more complex model structure with 13 growth-factors as predictors (Fig. 8). In this work, *Fagus sylvatica* was most strongly impacted by current year climatic conditions, particularly those of June to July of the year of ring formation (compare Fig. 5). Current-year temperature had a negative and soil moisture (from January to July) a positive effect on radial growth. In our work, predicted higher temperatures in June and July and decreased soil moisture from April to July are mainly responsible for a modelled moderate decline in ring width (-0.15 mm) in the RCP8.5 scenario (in comparison with the baseline scenario) till the end of the century (Fig. 10).

*Larix decidua* was sensitive to soil moisture levels - especially in June and July of the year of ring formation (Fig. 8). Also, there was a negative response to April to July temperature of the year before ring formation. Our long-term predictions reveal a distinct decline of annual ring width, especially for the far future (2081 – 2100) in the RCP8.5 scenario (-0.21 mm; Fig. 10).

*Picea abies* showed strong response to high soil moisture and low temperature in June and July of the year of ring formation (Fig. 8). We project a growth decline of −0.26 mm per annum until the end of the century (RCP8.5 vs. baseline; Fig. 10).

*Pinus nigra* showed reduced radial growth with a decline of the climatic water balance during June and July of the current and previous summers (Fig. 8). Our study indicates that *Pinus nigra* exhibits the most significant projected growth decline of all investigated species, with an average growth reduction of −0.29 mm by the end of the century (Fig. 10).

*Pinus sylvestris* was highly sensitive to drought, with significant responses to current year summer precipitation (July and August; Fig. 8). We model an average decline of the ring width residual of −0.21 mm until the end of this century (Fig. 10).

In our LME approach *Quercus cerris* exhibited a positive growth response to late summer and autumn temperatures (Fig. 8), making it the only species reacting positively to climate change (+0.12 mm per annum at the end of the century in the RCP8.5 scenario; Fig. 10). *Quercus petraea* was modelled with a minor decline of the mean residual width of – 0.09 mm.

## 4 Discussion

### 4.1 Predictions of phenological changes

For LU in *Fagus sylvatica* Wang et al. (2022) estimated an earlier onset of the growing season by 6.8 and 9 days (RCP4.5 and RCP8.5) until the end of the century. Their estimates lie well in the range of our predictions for LU (Fig. 3). The earlier onset of the growing season is expected to exacerbate water consumption at the beginning of this period, thereby increasing the drought risk, mainly in the first half of the growing season.

### 4.2 Growth factors

Water balance modelling points to rising annual transpiration sums by ~21%, until 2100 in the RCP8.5 scenario (Fig. 4). This result is comparable to prediction results reported by Misson et al. (2002): For Central European *Fagus sylvatica* forests in Belgium they reported a ~17% increase in transpiration, until the end of the 21st century, which in their study was associated with a temperature rise of ~2.8 °C. In our simulation, increased transpiration also leads to slightly drier soils: The transpiration effect is only partially mitigated by increased precipitation sums in the climate change scenarios, supporting hypothesis 1. Rising annual precipitation sums are primarily driven by rising winter precipitation. According to Haslinger et al. (2023) the climatic water balance may experience moderate increases during winter and spring, however, drought risk is expected to escalate in summer months.

### 4.3 Tree-ring residual modelling

Evaluating oxygen isotope ratios (δ^18^O) in tree-ring cellulose of *Acer pseudoplatanus* Battipaglia et al. (2008) demonstrated that this species is highly sensitive to spring precipitation, suggesting high vulnerability to current year water deficit. Our simulation points towards relevance of water availability during June and July of the actual year (number of drought days) and the year preceding ring formation (climatic water balance) current growth year (Fig. 8), pointing towards a moderate future decline of radial growth.

Radial growth of *Fagus sylvatica* was shown to be strongly linked to leaf photosynthesis, with the steepest increase of ring growth from May to June when the canopy reaches their maximum leaf mass area and photosynthetic rate (Čufar et al., 2008; Michelot et al., 2012). At a typical forest site in Slovenia, ring formation of *Fagus sylvatica* was also negatively affected by August maximum temperatures and positively affected by May and July precipitation (Čufar et al., 2008). In our work, predicted higher temperatures in June and July and decreased soil moisture from April to July (Fig. 8) are mainly responsible for the modelled future decline in radial growth.

Using the same dendrochronological database as this study, Winter Artusio et al. (2025) described *Larix decidua* as a tree species with favourable performance regarding drought stress resistance, resilience and recovery from drought years. This was due to the observed growth decline during the drought year being overcompensated in subsequent periods. Their approach evaluated growth performance (tree-ring width) in drought years in comparison to years before and after the event for four drought periods in the last 90 years. The observed post drought growth overcompensation of *Larix decidua* may be indirectly linked to the simultaneously observed growth depression of *Fagus sylvatica*: The species transpires substantially more during the growing season than admixed conifers (Berger et al., 2006), so a post-drought growth depression in *Fagus sylvatica* and the associated reduction in water uptake could increase soil water availability and thereby promote compensatory growth in *Larix decidua*. In contrast to current behaviour of *Larix decidua*, our long-term predictions show a distinct decline of annual ring width, especially for the far future (Fig. 10).

Our projections for *Picea abies* indicate a substantial decline in radial growth through the end of the century (Fig. 10), a result that aligns with recent literature. Declines at low and mid elevations are mainly driven by higher temperatures and reduced, more unevenly distributed, precipitation that increase summer soil water deficits (D’Andrea et al., 2023). The species’ shallow rooting - roughly 80% of fine roots in the upper 20–25 cm - renders it especially vulnerable to topsoil drying (Rosengren et al., 2006), and drought-weakened stands are more prone to secondary attacks by the spruce bark beetle (Dudík et al., 2021).

Our study indicates that *Pinus nigra* exhibits the most significant projected growth decline of all investigated species, with an average growth reduction of −0.29 mm by the end of the century (Fig. 10). We formulated hypothesis 3 that among coniferous species *Pinus nigra* exhibits the most favorable response regarding climate change. Our findings do not support that statement. The observed poor performance of *Pinus nigra* might be the result of two interacting factors: chronic drought stress and tip blight caused by the fungus *Diplodia sapinea*. The fungus can live symptomless as an endophyte or saprobe and become pathogenic under warm, wet spring conditions followed by summer drought, preferentially attacking trees already weakened by drought and thereby amplifying climate-driven decline (Schwanda et al., 2022).

According to Brichta et al. (2024), the growth of *Pinus sylvestris* is more sensitive to temperature than to total precipitation. In our work the species’ growth response was sensitive to the climatic water balance, transpiration sums, air temperature and days below REW 0.7 (Fig. 8). We modelled declining radial growth until the end of this century (Fig. 10). This projected decrease is consistent with observed dieback and a possible range contraction of *Pinus sylvestris* in parts of Central Europe (Klisz et al., 2023), and reflects both reductions in summer water availability and increased temperatures.

In Europe, the broad-leaved forests of lowland areas and lower elevations are predominantly dominated by two deciduous oak species: *Quercus petraea* and *Quercus robur*. While extensive research has focused on the climate-growth relationships of these species across various European regions, *Quercus cerris*, which frequently coexist and form mixed stands with the latter two, remains under-researched from a dendroclimatological perspective (Mészáros et al., 2022). *Quercus petraea* and *Quercus cerris*, both have been described as drought-tolerant species (Kunz et al., 2018). Móricz et al., (2021) found that for three Hungarian forest sites *Quercus petraea* and *Quercus cerris*, both heavily depend on summer water availability for radial growth. However, *Quercus cerris* shows greater drought sensitivity but higher recovery potential, while *Quercus petraea* displays lower growth plasticity and slower post-drought recovery. These contrasting strategies suggest *Quercus cerris* may gain competitive advantage under increasing drought stress (Winter Artusio et al., 2025).

Our study demonstrated clear differences in the climate sensitivity of *Quercus cerris* and *Quercus petraea*, suggesting that *Quercus cerris* may be better equipped for the anticipated climate change. It is the only species exhibiting a positive growth response to late summer and autumn temperatures (Fig. 8), making it the only species reacting positively to climate change (+0.12 mm per annum at the end of the century in the RCP8.5 scenario; Fig. 10). *Quercus petraea* displayed only a minor reduction in radial growth, making it the species with the second-best suitability for climate change conditions (Fig. 10).

Our results strongly support hypothesis 2: model selection consistently identified current-year June-July conditions as the dominant drivers of interannual variability in ring-width residuals across species, and within that window water-related variables such as soil moisture, climatic water balance and water-stress day counts were the most consistently predictors. Temperature in the target season tended to have negative effects. While previous-year conditions did influence growth in the simulations, their explanatory power was generally lower than that of current growing-season June-July factors.

Hypothesis 3 is supported in broad terms: most conifers in our study *(Picea abies, Pinus sylvestris, Larix decidua)* show stronger negative responses to elevated summer temperature and reduced summer soil moisture than the deciduous species, and - within deciduous trees - the *Quercus* species perform comparatively well. However, the specific expectation that *Pinus nigra* would be the relatively favourable conifer is not supported: *Pinus nigra* exhibits the largest projected decline. This exception highlights that species-level vulnerability cannot be inferred from climate tolerances alone; biotic threats, such as pathogenic fungi or bark beetles that preferentially attack already drought-stressed trees, might strongly affect the trees’ future performance.

## 5 Conclusion

In the future, the Vienna Woods are assumed to experience more frequent climatic extremes, such as heat waves and droughts, exacerbating the climatic pressure on the functioning of forest ecosystems. In our simulation, soils are predicted to become drier. The projected rise of MAP in the climate change scenarios does not fully compensate for the increased transpirative demand due to rising temperatures. Almost all investigated species are predicted to respond negatively to the projected climate conditions. For the far future (2081–2100) of the RCP8.5 scenario, average declines in the annual tree ring width residual (compared to baseline) are ranked as follows: *Pinus nigra*: −0.29 mm, *Larix decidua*: −0.27 mm, *Picea abies*: −0.26 mm, *Pinus sylvestris*: −0.21 mm, *Fagus sylvatica*: −0.15 mm, *Acer pseudoplatanus*: −0.14 mm, *Quercus petraea*: −0.09 mm and *Quercus cerris*: +0.12 mm. These findings clearly indicate that coniferous species are likely to exhibit greater vulnerability to climate change than deciduous species. The investigated oak species are displaying the most favorable response. Of all tree species tested, *Quercus cerris* was exceptional in exhibiting a predicted small increase in future radial growth attributed to a favorable response to higher temperatures during the late summer and the preceding autumn. This study assessed the current and future climate sensitivities of long-established tree species in the Vienna Woods. Local forest management - which is already setting the focus on the *Quercus* genus - should specifically consider the advantages that *Quercus cerris* might display over *Quercus petraea* to increase the overall resilience and sustainability of regional forest ecosystems in the face of climate change.

## Figures and Tables

**Table 1:** Optimized parameters for the age-width relationship. *p*_n_, *p*_s_,*w*_50_, and *p*_m_ describe the shape of the transition of the age-width relation curve. *w*_min_ denotes the assumed minimum ring width (see Section 2.2.1). N corresponds to the number of distinct ages (years) in the modelling of the age width relationship. Width was modelled on one assembled age timeline for each species. The low r^2^ value for *Acer pseudoplatanus* can be explained by the low number of seven sampled trees.

Species	*p*_n_	*p*_s_	*w*_50_	*w*_min_	*p*_m_	N	r^2^	adj. r^2^
*Acer pseudoplatanus*	1.5	0.00	1.1	0.7	218.5	127	0.31	0.28
*Fagus sylvatica*	2.3	0.00	1.7	0.9	218.5	203	0.96	0.96
*Larix decidua*	25.3	0.00	1.0	0.5	141.3	177	0.97	0.97
*Picea abies*	9.0	0.00	1.9	0.9	92.0	114	0.93	0.93
*Pinus nigra*	14.6	0.00	1.0	0.7	151.6	194	0.90	0.90
*Pinus sylvestris*	22.2	0.00	1.1	0.3	121.5	149	0.98	0.98
*Quercus cerris*	9.9	3.23	1.6	1.0	112.0	164	0.90	0.90
*Quercus petraea*	13.8	2.21	1.3	0.9	143.2	196	0.96	0.96

**Table 2:** Used data and performance of the LME model. Marginal r^2^ represents the portion of r^2^ which can be explained by fixed effects (growth factors) only. Conditional r^2^ comprises also the random effects (site) part. As *Acer pseudoplatanus* was only present at one site, the value for conditional r^2^ is not meaningful and therefore excluded. Due to reasons of consistency, *Acer pseudoplatanus’* marginal r^2^ is calculated analogous as for the other species.

Species	N sites	Start	End	N years	marg. r^2^	cond. r^2^
*Acer pseudoplatanus*	1	1933	2022	90	0.30	
*Fagus sylvatica*	63	1933	2022	90	0.19	0.48
*Larix decidua*	13	1933	2021	89	0.24	0.40
*Picea abies*	5	1933	2021	89	0.23	0.35
*Pinus nigra*	6	1933	2021	89	0.20	0.70
*Pinus sylvestris*	4	1933	2021	89	0.19	0.46
*Quercus cerris*	12	1933	2021	89	0.10	0.55
*Quercus petraea*	26	1933	2022	90	0.06	0.60

**Table 3:** Predictors of the LME model for all examined tree species.

*Species*	Growth Factor	Season	Est.	P
*Acer pseudoplatanus*	Climatic W.B.	Jun, Jul (-1)	0.07787	0.00285
*Acer pseudoplatanus*	Days below REW 0.4	Jun, Jul (act.)	-0.15272	0.00000
*Acer pseudoplatanus*	Intercept		-0.05678	0.44713
*Fagus sylvatica*	Mean Temp.	Apr, May, (-2)	-0.03708	0.00000
*Fagus sylvatica*	Climatic W.B.	Oct - Dec (-2)	-0.06630	0.00000
*Fagus sylvatica*	Mean Temp.	Jan - Mar (-1)	0.01716	0.00021
*Fagus sylvatica*	Days below REW 0.7	Apr, May, (-1)	-0.08133	0.00000
*Fagus sylvatica*	Days below REW 0.4	Aug, Sep (-1)	-0.04896	0.00000
*Fagus sylvatica*	Climatic W.B.	Oct - Dec (-1)	-0.07195	0.00000
*Fagus sylvatica*	Mean Soil Moisture	Jan - Mar (act.)	0.04464	0.00000
*Fagus sylvatica*	Mean Temp.	Jan - Mar (act.)	0.04593	0.00000
*Fagus sylvatica*	Mean Soil Moisture	Apr, May, (act.)	0.09575	0.00000
*Fagus sylvatica*	Mean Soil Moisture	Jun, Jul (act.)	0.09289	0.00000
*Fagus sylvatica*	Mean Temp.	Jun, Jul (act.)	-0.09922	0.00000
*Fagus sylvatica*	Days below REW 0.4	Aug, Sep (act.)	-0.02148	0.00026
*Fagus sylvatica*	Mean Temp.	Aug, Sep (act.)	0.07332	0.00000
*Fagus sylvatica*	Intercept		0.09740	0.00947
*Larix decidua*	Days below REW 0.4	Aug, Sep (-2)	-0.03861	0.00006
*Larix decidua*	Mean Temp.	Jan - Mar (-1)	0.03328	0.00020
*Larix decidua*	Mean Temp.	Apr, May, (-1)	-0.06973	0.00000
*Larix decidua*	Days below REW 0.7	Jun, Jul (-1)	-0.04076	0.00141
*Larix decidua*	Mean Temp.	Jun, Jul (-1)	-0.06490	0.00000
*Larix decidua*	Transpiration Deficit	Aug, Sep (-1)	-0.05817	0.00001
*Larix decidua*	Transpiration Deficit	Oct - Dec (-1)	-0.03364	0.00068
*Larix decidua*	Mean Temp.	Oct - Dec (-1)	0.04852	0.00009
*Larix decidua*	Mean Soil Moisture	Jun, Jul (act.)	0.08654	0.00000
*Larix decidua*	Intercept		-0.03972	0.43233
*Picea abies*	Climatic W.B.	Jun, Jul (-2)	0.06467	0.00305
*Picea abies*	Climatic W.B.	Oct - Dec (-2)	-0.07969	0.00000
*Picea abies*	Mean Temp.	Jun, Jul (-1)	-0.09051	0.00044
*Picea abies*	Mean Soil Moisture	Aug, Sep (-1)	0.07322	0.00047
*Picea abies*	Climatic W.B.	Oct - Dec (-1)	-0.04843	0.00331
*Picea abies*	Mean Temp.	Jan - Mar (act.)	0.07482	0.00032
*Picea abies*	Mean Soil Moisture	Jun, Jul (act.)	0.14294	0.00000
*Picea abies*	Intercept		-0.09007	0.33502
*Pinus nigra*	Transpiration Deficit	Oct - Dec (-2)	-0.04509	0.00001
*Pinus nigra*	Mean Soil Moisture	Jan - Mar (-1)	-0.02844	0.00064
*Pinus nigra*	Climatic W.B.	Jun, Jul (-1)	0.06264	0.00000
*Pinus nigra*	Mean Temp.	Aug, Sep (-1)	-0.07415	0.00000
*Pinus nigra*	Mean Temp.	Jan - Mar (act.)	0.06290	0.00000
*Pinus nigra*	Climatic W.B.	Jun, Jul (act.)	0.11236	0.00000
*Pinus nigra*	Mean Temp.	Jun, Jul (act.)	-0.03799	0.00225
*Pinus nigra*	Intercept		0.06780	0.60535
*Pinus sylvestris*	Mean Temp.	Apr, May, (-1)	-0.05677	0.00000
*Pinus sylvestris*	Days below REW 0.7	Aug, Sep (-1)	-0.03525	0.00044
*Pinus sylvestris*	Mean Temp.	Jan - Mar (act.)	0.05996	0.00000
*Pinus sylvestris*	Transp. Sum	Apr, May, (act.)	-0.08309	0.00000
*Pinus sylvestris*	Climatic W.B.	Jun, Jul (act.)	0.06759	0.00000
*Pinus sylvestris*	Intercept		-0.02137	0.79019
*Quercus cerris*	Mean Temp.	Oct - Dec (-2)	0.04738	0.00000
*Quercus cerris*	Days below REW 0.4	Aug, Sep (-1)	-0.02742	0.00066
*Quercus cerris*	Transpiration Deficit	Oct - Dec (-1)	-0.02638	0.00058
*Quercus cerris*	Mean Temp.	Oct - Dec (-1)	0.04716	0.00000
*Quercus cerris*	Mean Temp.	Apr, May, (act.)	-0.05749	0.00000
*Quercus cerris*	Mean Soil Moisture	Jun, Jul (act.)	0.06259	0.00000
*Quercus cerris*	Mean Temp.	Jun, Jul (act.)	-0.04537	0.00014
*Quercus cerris*	Mean Temp.	Aug, Sep (act.)	0.06196	0.00000
*Quercus cerris*	Intercept		0.03516	0.65008
*Quercus petraea*	Transpiration Deficit	Apr, May, (-2)	-0.02077	0.00034
*Quercus petraea*	Mean Temp.	Jun, Jul (-2)	-0.04490	0.00000
*Quercus petraea*	Days below REW 0.7	Jan - Mar (-1)	0.01712	0.00004
*Quercus petraea*	Mean Temp.	Jan - Mar (-1)	0.02034	0.00005
*Quercus petraea*	Mean Temp.	Oct - Dec (-1)	0.03834	0.00000
*Quercus petraea*	Transp. Sum	Apr, May, (act.)	-0.02526	0.00014
*Quercus petraea*	Mean Soil Moisture	Jun, Jul (act.)	0.07746	0.00000
*Quercus petraea*	Days below REW 0.4	Aug, Sep (act.)	-0.02133	0.00019
*Quercus petraea*	Intercept		0.09332	0.13395

## Data Availability

Data will be made available on request.
